# Supplementary material for: The mechanism of action of auranofin analogs in B. cenocepacia revealed by chemogenomic profiling
Source: Microbiol Spectr. 2024 Jan 11;12(2):e03201-23. doi: 10.1128/spectrum.03201-23 (PMC10846046; doi:10.1128/spectrum.03201-23)
Supplement: Supplemental Tables and Figures — Tables S1-S4; Figures S1-S8. [file spectrum.03201-23-s0003.docx]

Supplemental material for:

Maydaniuk et al., The mechanism of action of auranofin analogs in *B. cenocepacia* revealed by chemogenomic profiling

**Tables S1-S4** are given here

**Figures S1-S8** are included here

**Supplemental Data 1**: Given as a separate Εxcel file

**Table S1.** Plasmids used in this study

| **Plasmids** | **Features** | **Source** |
| --- | --- | --- |
| pDAI-SceI-*sacB* | pDAI-SceI expressing *sacB* | [1,2] |
| pGPI-SceI | ori_R6K_ Tmp^r^ mob^+^ carries I-SceI cut site | [3] |
| pSCB2-sgRNA | Template for pgRNA created by inverse PCR; derived from  pSCrhaB2-sgRNA by inverse  PCR to remove *rhaS, rhaR,* and  P*rhaB* | [4] |
| pgRNA-non-target | Derived from pSCB2-sgRNA; random 20 nt sequence added as gRNA binding region by inverse PCR | [4] |
| pGPI-SceI-*gor* | pGPI-SceI with a fusion of approximately 350 bp regions immediately upstream and downstream of *gor* cloned into *Xba*I and *Xma*I sites. Enables deletion of *gor* gene. | This study |
| pGPI-SceI-*gshA1* | pGPI-SceI with a fusion of approximately 350 bp regions immediately upstream and downstream of *gshA1* cloned into *Xba*I and *Xma*I sites. Enables deletion of *gshA1* gene. | This study |
| pGPI-SceI-*gshA2* | pGPI-SceI with a fusion of approximately 350 bp regions immediately upstream and downstream of *gshA2* cloned into *Xba*I and *Xma*I sites. Enables deletion of *gshA2* gene. | This study |
| pGPI-SceI-*gshB* | pGPI-SceI with a fusion of approximately 350 bp regions immediately upstream and downstream of *gshB* cloned into *Xba*I and *Xma*I sites. Enables deletion of *gshB* gene. | This study |
| pGPI-SceI-*grxC* | pGPI-SceI with a fusion of approximately 350 bp regions immediately upstream and downstream of *grxC* cloned into *Xba*I and *Xma*I sites. Enables deletion of *grxC* gene. | This study |
| pGPI-SceI-*grxD* | pGPI-SceI with a fusion of approximately 350 bp regions immediately upstream and downstream of *grxD* cloned into *Xba*I and *Xma*I sites. Enables deletion of *grxD* gene. | This study |
| pgRNA-*trxB* | pSCB2-sgRNA expressing sgRNA targeting *trxB* | This study |
| pgRNA-*rodA* | pSCB2-sgRNA expressing sgRNA targeting *rodA* | This study |
| pET-24b-TrxB | pET-24b+ containing *trxB* gene from *B. cenocepacia* K56-2 | This study |
| pET-22-GOR | pET-22+ containing *gor* gene from *B. cenocepacia* K56-2 | This study |
| pgRNA-*gshA1gshB* | pSCB2-sgRNA expressing sgRNA targeting *gshA1 gshB* operon | This study |

**Table S2**. Strains and mutants used in this study

| Strain | Features | Source |
| --- | --- | --- |
| *B. cenocepacia* K56-2 | ET12 lineage CF clinical isolate from Toronto | [6] |
| *B. cenocepacia* K56-2::dCas9 | Derived from K56-2; pAH-CTX1rhadCas9 integrated at *attB* site; clean deletion of plasmid accessory genes | [4] |
| *B. cenocepacia* K56-2::dCas9 pgRNA-non-target | Non-targeting CRISPRi mutant | [4] |
| *B. cenocepacia* K56-2 Δ*gshA1* | Unmarked gene deletion of gshA1, K562_ RS03750 | This study |
| *B. cenocepacia* K56-2 Δ*gshA2* | Unmarked gene deletion of gshA2, K562_ RS02270 | This study |
| *B. cenocepacia* K56-2 Δ*gshB* | Unmarked gene deletion of gshB, K562_ RS03755 | This study |
| *B. cenocepacia* K56-2 Δ*grxC* | Unmarked gene deletion of grxC, K562_ RS03795 | This study |
| *B. cenocepacia* K56-2 Δ*grxD* | Unmarked gene deletion of grxD, K562_ RS01940 | This study |
| *B. cenocepacia* K56-2 Δ*gor* | Unmarked gene deletion of gor, K562_ RS03010 | This study |

**Table S3**. Primers used in this study

| **Primer Number** | **Sequence (5’ to 3’)** | **Notes** |
| --- | --- | --- |
| 848 | CCGCCAGGCAAATTCTGTTT | Reverse primer for colony PCR, all gRNAs |
| 1092 | ACTAGTATTATACCTAGGACTGAGCTAGC | Reverse primer, all gRNAs |
| 1409 | GGCTTATGTCAACTGGGTTCG | Forward primer for general colony PCR of sgRNA plasmids |
| 2163 | CAAGCAGAAGACGGCATACGAGATGCTTACGGACGTCTCGTGG  GCTCGGAGATGTGTATAAGAGACAGGTCGACCTGCAGCGTACG | BarSeq primer with i7 adapter; UDP0089 |
| 2164 | CAAGCAGAAGACGGCATACGAGATCGCTTGAAGTGTCTCGT  GGGCTCGGAGATGTGTATAAGAGACAGGTCGACCTGCAGCGTACG | BarSeq primer with i5 adapter; UDP0089 |
| 2171 | AATGATACGGCGACCACCGAGATCTACACCGTGTATCTTT  CGTCGGCAGCGTCAGATGTGTATAAGAGACAGGATGTCCACGAGGTCTCT | BarSeq primer with i7 adapter; UDP0090 |
| 2172 | AATGATACGGCGACCACCGAGATCTACACGAACCATGAATCGT  CGGCAGCGTCAGATGTGTATAAGAGACAGGATGTCCACGAGGTCTCT | BarSeq primer with i5 adapter; UDP0090 |
| 2224 | CAAGCAGAAGACGGCATACGAGATGTTCCGCAGGGTCTCGTGGGC  TCGGAGATGTGTATAAGAGACAGGTCGACCTGCAGCGTACG | BarSeq primer with i7 adapter; UDP0073 |
| 2225 | AATGATACGGCGACCACCGAGATCTACACATCATAGGCTTCGTCGGCAG  CGTCAGATGTGTATAAGAGACAGGATGTCCACGAGGTCTCT | BarSeq primer with i5 adapter; UDP0073 |
| 2226 | CAAGCAGAAGACGGCATACGAGATACCTTATGAAGTCTCGTGGGCTC  GGAGATGTGTATAAGAGACAGGTCGACCTGCAGCGTACG | BarSeq primer with i7 adapter; UDP0074 |
| 2227 | AATGATACGGCGACCACCGAGATCTACACTGTTAGAAGGTCGTCGGCA  GCGTCAGATGTGTATAAGAGACAGGATGTCCACGAGGTCTCT | BarSeq primer with i5 adapter; UDP0074 |
| 2228 | CAAGCAGAAGACGGCATACGAGATCGCTGCAGAGGTCTCGTGGGC  TCGGAGATGTGTATAAGAGACAGGTCGACCTGCAGCGTACG | BarSeq primer with i7 adapter; UDP0075 |
| 2229 | AATGATACGGCGACCACCGAGATCTACACGATGGATGTATCGTC  GGCAGCGTCAGATGTGTATAAGAGACAGGATGTCCACGAGGTCTCT | BarSeq primer with i5 adapter; UDP0075 |
| 2230 | CAAGCAGAAGACGGCATACGAGATGTAGAGTCAGGTCTCGT  GGGCTCGGAGATGTGTATAAGAGACAGGTCGACCTGCAGCGTACG | BarSeq primer with i7 adapter; UDP0076 |
| 2231 | AATGATACGGCGACCACCGAGATCTACACACGGCCGTCATC  GTCGGCAGCGTCAGATGTGTATAAGAGACAGGATGTCCACGAGGTCTCT | BarSeq primer with i5 adapter; UDP0076 |
| 2232 | CAAGCAGAAGACGGCATACGAGATGGATACCAGAGTCTCGT  GGGCTCGGAGATGTGTATAAGAGACAGGTCGACCTGCAGCGTACG | BarSeq primer with i7 adapter; UDP0077 |
| 2233 | AATGATACGGCGACCACCGAGATCTACACCGTTGCTTACTC  GTCGGCAGCGTCAGATGTGTATAAGAGACAGGATGTCCACGAGGTCTCT | BarSeq primer with i5 adapter; UDP0077 |
| 2234 | CAAGCAGAAGACGGCATACGAGATCGCACTAATGGTC  TCGTGGGCTCGGAGATGTGTATAAGAGACAGGTCGACCTGCAGCGTACG | BarSeq primer with i7 adapter; UDP0078 |
| 2235 | AATGATACGGCGACCACCGAGATCTACACTGACTACATAT  CGTCGGCAGCGTCAGATGTGTATAAGAGACAGGATGTCCACGAGGTCTCT | BarSeq primer with i5 adapter; UDP0078 |
| 2236 | CAAGCAGAAGACGGCATACGAGATTCCTGACCGTGTCTC  GTGGGCTCGGAGATGTGTATAAGAGACAGGTCGACCTGCAGCGTACG | BarSeq primer with i7 adapter; UDP0079 |
| 2237 | AATGATACGGCGACCACCGAGATCTACACCGGCCTCGTTTC  GTCGGCAGCGTCAGATGTGTATAAGAGACAGGATGTCCACGAGGTCTCT | BarSeq primer with i5 adapter; UDP0079 |
| 2238 | CAAGCAGAAGACGGCATACGAGATCTGGCTTGCCGTCTC  GTGGGCTCGGAGATGTGTATAAGAGACAGGTCGACCTGCAGCGTACG | BarSeq primer with i7 adapter; UDP0080 |
| 2239 | AATGATACGGCGACCACCGAGATCTACACCAAGCATCCGTCG  TCGGCAGCGTCAGATGTGTATAAGAGACAGGATGTCCACGAGGTCTCT | BarSeq primer with i5 adapter; UDP0080 |
| 2240 | CAAGCAGAAGACGGCATACGAGATACCAGCGACAGTCTC  GTGGGCTCGGAGATGTGTATAAGAGACAGGTCGACCTGCAGCGTACG | BarSeq primer with i7 adapter; UDP0081 |
| 2241 | AATGATACGGCGACCACCGAGATCTACACTCGTCTGACTTCGT  CGGCAGCGTCAGATGTGTATAAGAGACAGGATGTCCACGAGGTCTCT | BarSeq primer with i5 adapter; UDP0081 |
| 2242 | CAAGCAGAAGACGGCATACGAGATTTGTAACGGTGTCTCGTGG  GCTCGGAGATGTGTATAAGAGACAGGTCGACCTGCAGCGTACG | BarSeq primer with i7 adapter; UDP0082 |
| 2243 | AATGATACGGCGACCACCGAGATCTACACCTCATAGCGATCGTCGG  CAGCGTCAGATGTGTATAAGAGACAGGATGTCCACGAGGTCTCT | BarSeq primer with i5 adapter; UDP0082 |
| 2244 | CAAGCAGAAGACGGCATACGAGATGTAAGGCATAGTCTCGTGGGC  TCGGAGATGTGTATAAGAGACAGGTCGACCTGCAGCGTACG | BarSeq primer with i7 adapter; UDP0083 |
| 2245 | AATGATACGGCGACCACCGAGATCTACACAGACACATTATCGTCG  GCAGCGTCAGATGTGTATAAGAGACAGGATGTCCACGAGGTCTCT | BarSeq primer with i5 adapter; UDP0083 |
| 2246 | CAAGCAGAAGACGGCATACGAGATGTCCACTTGTGTCTCGTGGGC  TCGGAGATGTGTATAAGAGACAGGTCGACCTGCAGCGTACG | BarSeq primer with i7 adapter; UDP0084 |
| 2247 | AATGATACGGCGACCACCGAGATCTACACGCGCGATGTTTCGTCGG  CAGCGTCAGATGTGTATAAGAGACAGGATGTCCACGAGGTCTCT | BarSeq primer with i5 adapter; UDP0084 |
| 2248 | CAAGCAGAAGACGGCATACGAGATTTAGGTACCAGTCTCGTGG  GCTCGGAGATGTGTATAAGAGACAGGTCGACCTGCAGCGTACG | BarSeq primer with i7 adapter; UDP0085 |
| 2249 | AATGATACGGCGACCACCGAGATCTACACCATGAGTACTTCGT  CGGCAGCGTCAGATGTGTATAAGAGACAGGATGTCCACGAGGTCTCT | BarSeq primer with i5 adapter; UDP0085 |
| 2250 | CAAGCAGAAGACGGCATACGAGATGGAATTCCAAGTCTCGTGGGC  TCGGAGATGTGTATAAGAGACAGGTCGACCTGCAGCGTACG | BarSeq primer with i7 adapter; UDP0086 |
| 2251 | AATGATACGGCGACCACCGAGATCTACACACGTCAATACTCGTCGG  CAGCGTCAGATGTGTATAAGAGACAGGATGTCCACGAGGTCTCT | BarSeq primer with i5 adapter; UDP0086 |
| 2252 | CAAGCAGAAGACGGCATACGAGATCATGTAGAGGGTCTCGTGGG  CTCGGAGATGTGTATAAGAGACAGGTCGACCTGCAGCGTACG | BarSeq primer with i7 adapter; UDP0087 |
| 2253 | AATGATACGGCGACCACCGAGATCTACACGATACCTCCTTCGTCG  GCAGCGTCAGATGTGTATAAGAGACAGGATGTCCACGAGGTCTCT | BarSeq primer with i5 adapter; UDP0087 |
| 2254 | CAAGCAGAAGACGGCATACGAGATTACACGCTCCGTCTCGTGGG  CTCGGAGATGTGTATAAGAGACAGGTCGACCTGCAGCGTACG | BarSeq primer with i7 adapter; UDP0088 |
| 2255 | AATGATACGGCGACCACCGAGATCTACACATCCGTAAGTTCG  TCGGCAGCGTCAGATGTGTATAAGAGACAGGATGTCCACGAGGTCTCT | BarSeq primer with i5 adapter; UDP0088 |
| 2303 | GCATTGGGATCCCATGTCCACGCCCAAACACG | Forward primer to amplify *trxB* for insertion into pET-24+ |
| 2304 | TGCGTAAAGCTTCAGGCTTTCGAGGTAGCGC | Reverse primer to amplify *trxB* for insertion into pET-24+ |
| 2315 | CCCGCGAAATTAATACGACT | Colony PCR primer for pET-24+ insertions |
| 2316 | GTTAGCAGCCGGATCTCA | Colony PCR primer for pET-24+ insertions |
| 2420 | CGCGAGCATCAGCGATGCGCGTTTT  AGAGCTAGAAATAGCAAGTTAAAATAAGGC | iPCR primer sgRNA to create CRISPRi mutant targeting *lolA* (K562_RS14590) |
| 2440 | GTGGCCGTCCGCTTGCTGCGGTTTTA  GAGCTAGAAATAGCAAGTTAAAATAAGGC | iPCR primer sgRNA to create CRISPRi mutant targeting *shc* (K562_RS31520) |
| 2449 | CGCGGTCGCGATTCGGGAACGTTTTAG  AGCTAGAAATAGCAAGTTAAAATAAGGC | iPCR primer sgRNA to create CRISPRi mutant targeting *bpeEF-oprC* operon (K562_RS30120) |
| 2827 | AGCTTCGAGTTTATCGCCCAGTTTTA  GAGCTAGAAATAGCAAGTTAAAATAAGGC | iPCR primer sgRNA to create CRISPRi mutant targeting *ahpC* (K562_RS08590) |
| 2839 | TGCCTGCGGTGTGGTTGAACGTTTTA  GAGCTAGAAATAGCAAGTTAAAATAAGGC | iPCR primer sgRNA to create CRISPRi mutant targeting *katG* (K562_RS15960) |
| 2874 | GCTGGAAAAGATGGCACTGC | Colony PCR primer for *gor* deletion |
| 2875 | ATTATCTAGAGACGCAGCTCATCAATCGCC | Forward primer for amplifying upstream region for *grxC* deletion |
| 2876 | ATTAAAGCTTCTGCATGCAATACGGACACAC | Reverse primer for amplifying upstream region for *grxC* deletion |
| 2877 | ATTAAAGCTTCCTCGTGCCGCTTCTGC | Forward primer for amplifying downstream region for *grxC* deletion |
| 2878 | ATTACCCGGGGAAAATGCCGGCCTGCTTC | Reverse primer for amplifying downstream region for *grxC* deletion |
| 2879 | CGTCCGGAATGTTGCGAATG | Colony PCR primer for *grxC* deletion |
| 2880 | CCAATTACACGAACCTGGCC | Colony PCR primer for *grxC* deletion |
| 2881 | ATTATCTAGACGCATCTCGCCCAGGG | Forward primer for amplifying upstream region for *grxD* deletion |
| 2882 | ATTAAAGCTTGCTTGATACGTTGTTGGGTGTC | Reverse primer for amplifying upstream region for *grxD* deletion |
| 2883 | ATTAAAGCTTCTCGGACATCATGATGGAGATGT | Forward primer for amplifying downstream region for *grxD* deletion |
| 2884 | ATTACCCGGGCGATCACCATGCCATCGG | Reverse primer for amplifying downstream region for *grxD* deletion |
| 2885 | GCAACCCGCCGTACATCG | Colony PCR primer for *grxD* deletion |
| 2886 | CGAGGTTGAACGGCGTTTC | Colony PCR primer for *grxD* deletion |
| 2887 | ATTATCTAGAGCTGATCACGCTGGTCTACA | Forward primer for amplifying upstream region for *gshA1* deletion |
| 2888 | ATTAAAGCTTGGAACCATGAATCACTCGCGAG | Reverse primer for amplifying upstream region for *gshA1* deletion |
| 2889 | ATTAAAGCTTCGATCGAACTGGAAAAGACCG | Forward primer for amplifying downstream region for *gshA1* deletion |
| 2890 | ATTACCCGGGCGTCACGTACTCCATGTCGAAC | Reverse primer for amplifying downstream region for *gshA1* deletion |
| 2891 | CGTATTCGCGGTCAAGGAC | Colony PCR primer for *gshA1* deletion |
| 2892 | CTTGTTGAACACGCGTGC | Colony PCR primer for *gshA1* deletion |
| 2893 | ATTATCTAGAGTGCAGGAAGGCGTCTACAC | Forward primer for amplifying upstream region for *gshB* deletion |
| 2894 | ATTAAAGCTTGAATGTCCATGCGGGTCCTG | Reverse primer for amplifying upstream region for *gshB* deletion |
| 2895 | ATTAAAGCTTGAGATCATGGAGCAGACGGG | Forward primer for amplifying downstream region for *gshB* deletion |
| 2896 | ATTACCCGGGGGATCCCGGCCATGTCAGAT | Reverse primer for amplifying downstream region for *gshB* deletion |
| 2897 | CGATACAGTCCCGCAGC | Colony PCR primer for *gshB* deletion |
| 2898 | GAGATCGGCCGCATGAC | Colony PCR primer for *gshB* deletion |
| 2899 | ATTATCTAGAGTGCCGGCGTGACGTG | Forward primer for amplifying upstream region for *gshA2* deletion |
| 2900 | ATTAAAGCTTCATGGTGTTCGACATGGAGAC | Reverse primer for amplifying upstream region for *gshA2* deletion |
| 2901 | ATTAAAGCTTCCTATACGCTGAACCGCTTCA | Forward primer for amplifying downstream region for *gshA2* deletion |
| 2902 | ATTACCCGGGGAAGACGATGAAACACGCG | Reverse primer for amplifying downstream region for *gshA2* deletion |
| 2903 | GCTCGCCAGCCAGAAG | Colony PCR primer for *gshA2* deletion |
| 2904 | GCATCGTTTCCGCCCG | Colony PCR primer for *gshA2* deletion |
| 3002 | ATTACATATGATGGATTTCGACTACGACCTGTTC | Forward primer for GOR insertion into pET-22+ |
| 3006 | GACACGATCGGCATTCATC | Colony PCR for GOR insertion into pET-22+ |
| 3008 | CGCGTAACCACCACACCC | Colony PCR for GOR insertion into pET-22+ |
| 3122 | GAACGTCGTGGGTGAAATCGC | Colony PCR primer for *gor* deletion |
| 3123 | ATTATCTAGAGGCATGCTCGAGAAGAAGGAC | Forward primer for amplifying upstream region for *gor* deletion |
| 3124 | ATTAAAGCTTGGTCGTAGTCGAAATCCATCGC | Reverse primer for amplifying upstream region for *gor* deletion |
| 3125 | ATTAAAGCTTCCGATATCGACGCGGAAGA | Forward primer for amplifying downstream region for *gor* deletion |
| 3126 | ATTACCCGGGGGGGCACACGCACGC | Reverse primer for amplifying downstream region for *gor* deletion |
| 3136 | CACCTCGTTACCGCGTTGAAGTTTTAGAGCTAGAAATAGCAAGTTAAAATAAGGC | iPCR primer sgRNA to create CRISPRi mutant targeting *gshA1* and *gshB* (K562-RS03750 and K562_RS03755) |
| 3385 | TCGTACAAGCTTCTTCTGCCGCATCGTTACG | Reverse primer for GOR insertion into pET-22+ |

Table S4 Correlation coefficients across replicates

| Condition | Comparison | P_Rsquared | S_Rsquared | L_Rsquared |
| --- | --- | --- | --- | --- |
| MS-40 | R2 vs R1 | 0.39939385 | 0.4083684 | 0.39929617 |
| MS-40 | R3 vs R1 | 0.39125067 | 0.41283821 | 0.39105594 |
| MS-40 | R3 vs R2 | 0.40353638 | 0.42282533 | 0.40296908 |
| MS-40S | R2 vs R1 | 0.40392028 | 0.41623961 | 0.40172874 |
| MS-40S | R3 vs R1 | 0.4183166 | 0.41004143 | 0.41816966 |
| MS-40S | R3 vs R2 | 0.40428574 | 0.43607088 | 0.40278064 |
| As2O3 | R2 vs R1 | 0.28251276 | 0.31574629 | 0.28238944 |
| As2O3 | R3 vs R1 | 0.21687959 | 0.24647649 | 0.21661572 |
| As2O3 | R3 vs R2 | 0.30604211 | 0.34713015 | 0.30598202 |
| Dia | R2 vs R1 | 0.31852636 | 0.3447956 | 0.31826601 |
| Dia | R3 vs R1 | 0.35569607 | 0.36416315 | 0.35546362 |
| Dia | R3 vs R2 | 0.31131656 | 0.32273661 | 0.31075773 |
| DMSO | R2 vs R1 | 0.35593126 | 0.39357624 | 0.3551871 |
| DMSO | R3 vs R1 | 0.37655507 | 0.39757142 | 0.37648226 |
| DMSO | R3 vs R2 | 0.37460709 | 0.41045452 | 0.37421595 |
| H2O2 | R2 vs R1 | 0.31597828 | 0.35791605 | 0.31574631 |
| H2O2 | R3 vs R1 | 0.36036042 | 0.39010731 | 0.35996184 |
| H2O2 | R3 vs R2 | 0.30022819 | 0.34749326 | 0.30020372 |
| Para | R2 vs R1 | 0.3509452 | 0.39330833 | 0.35045932 |
| Para | R3 vs R1 | 0.30558972 | 0.34726794 | 0.30551982 |
| Para | R3 vs R2 | 0.29094457 | 0.35496047 | 0.290223 |

R1, R2, and R3 correspond to biological replicate 1, 2 and 3, respectively.

**
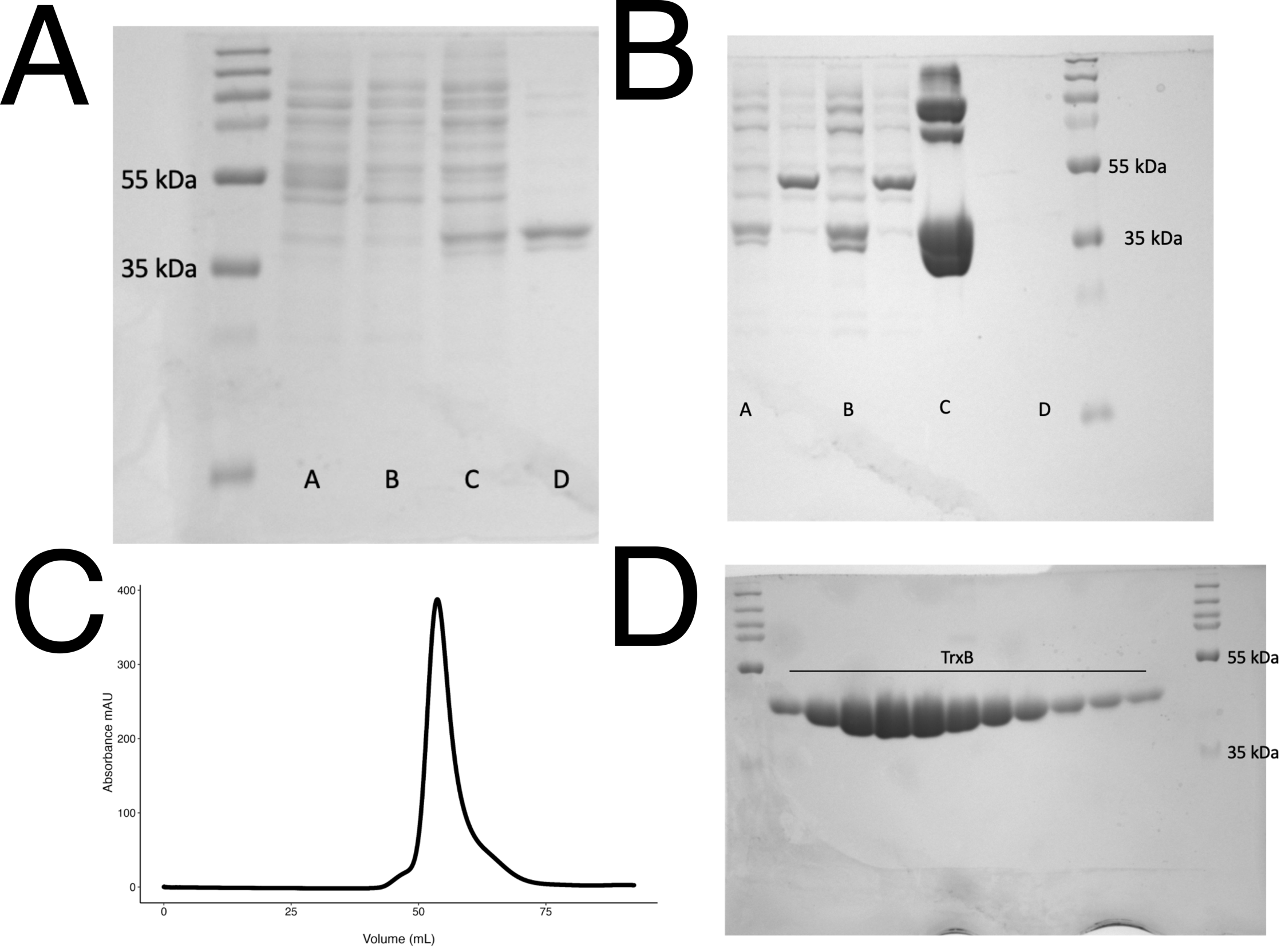
**

**Figure S1. Purification of TrxB. (A)** *E. coli* containing pET-24b+ (empty vector) and pET-24b-TrxB were grown until an OD of 0.6, then induction was started by adding 1 mM IPTG for half the samples. Induction was done for three hours. Resulting cultures were pelleted and resuspended in lysis buffer: 50 mM TRIS, 500 mM NaCl, 25 mM imidazole, pH 7.5. Lysis was done using sonication, and after sonication, soluble fraction was collected by centrifugation and ran on a 10% acrylamide gel. (A) pET-24b+ empty vector without induction. (B) pET-24b+ empty vector with induction. (C) pET-24b-TrxB without induction. (D) pET-24b-TrxB with induction. **(B)** *E. coli* containing pET-24b-TrxB were grown until an OD of 0.6, and then induction was started by adding 1 mM IPTG for half the samples. Induction was done for three hours. Resulting cultures were pelleted and resuspended in lysis buffer: 50 mM TRIS, 500 mM NaCl, 25 mM imidazole, pH 7.5. Lysis was done using an emulsiflex, and after the soluble fraction was collected by centrifugation ran through a nickel affinity column. (A) pET-24b-TrxB flow through. (B) pET-24b-TrxB wash. (C) pET-24b-TrxB elution. (D) pET-24b-TrxB second wash. **(C)** After elution, the TrxB protein was concentrated, and the elution buffer was replaced with a gel filtration buffer (20 mM Tris pH 7.5, 150 mM sodium chloride, and 1 mM β-ME). The concentrated protein was then run through an AKTA for SEC. **(D)** After elution, SEC and the resulting fractions were run on a 10% acrylamide gel.

**
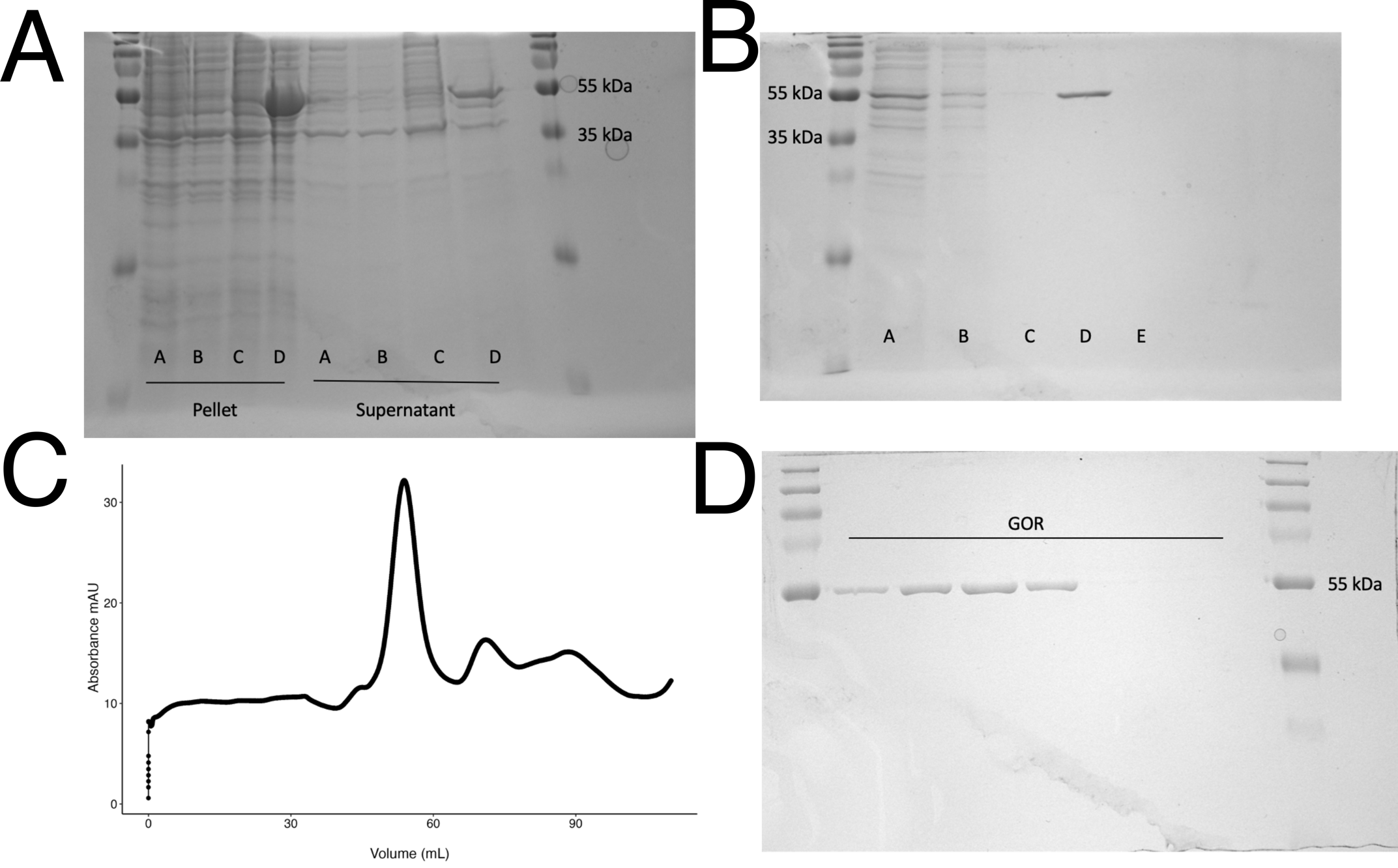
**

**Figure S2. Purification of GOR. (A)** *E. coli* containing pET-22 (empty vector) and pET-22-GOR were grown until an OD of 0.6, and then induction was started by adding 1 mM IPTG for half the samples. Induction was done for three hours. Resulting cultures were pelleted and resuspended in lysis buffer: 50 mM TRIS, 500 mM NaCl, 25 mM imidazole, pH 7.5. Lysis was done using sonication, and after sonication, soluble fraction was collected by centrifugation and ran on a 10% acrylamide gel. (A) pET-22 empty vector without induction. (B) pET-22 empty vector with induction. (C) pET-22-GOR without induction. (D) pET-22-GOR with induction. **(B)** *E. coli* containing pET-22-GOR was grown until an OD of 0.6, and then induction was started by adding 1 mM IPTG for half the samples. Induction was done for three hours. Resulting cultures were pelleted and resuspended in lysis buffer: 50 mM TRIS, 500 mM NaCl, 25 mM imidazole, pH 7.5. Lysis was done using an emulsiflex, and after the soluble fraction was collected by centrifugation ran through a nickel affinity column. (A) pET-22-GOR flow through. (B) pET-22-GOR wash. (C) pET-22-GOR wash. (D) pET-22-GOR elution. (E) pET-22-GOR wash after elution. **(C)** After elution, the GOR protein was concentrated, and the elution buffer was replaced with a gel filtration buffer (20 mM Tris pH 7.5, 150 mM sodium chloride, and 1 mM β-ME). The concentrated protein was then run through an AKTA for SEC. **(D)** After elution, SEC and the resulting fractions were run on a 10% acrylamide gel.


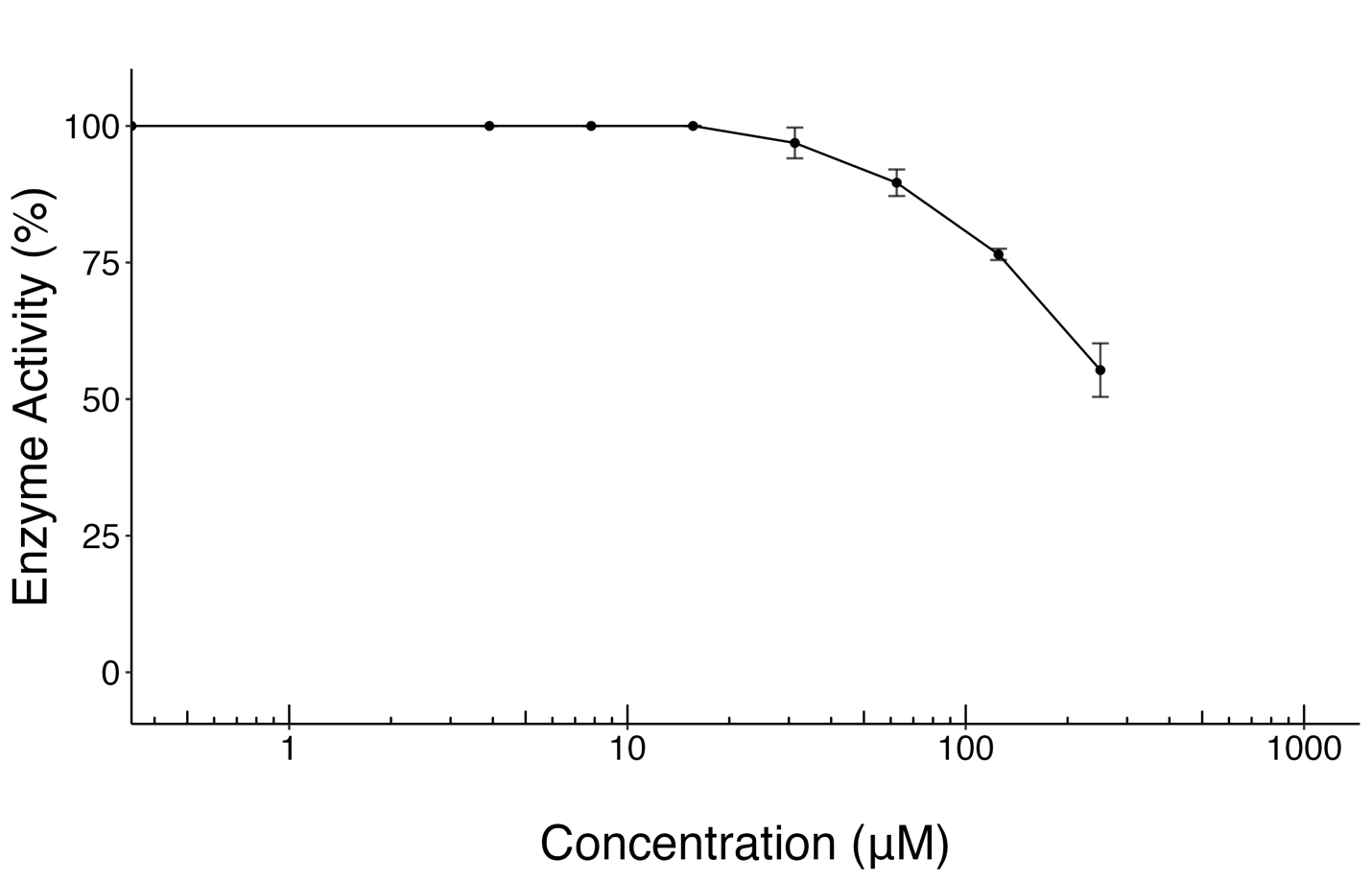


**Figure S3. 2-AAPA inhibits GOR from *B. cenocepacia* K56-2**. Purified GOR from *B. cenocepacia* K56-2 in the presence of substrate, oxidized glutathione is reduced to reduced glutathione, with NADPH as a source of electrons. This reaction tracks the usage of NADPH, which is detectable at 340 nm. Absorbance was measured every 5 minutes for 3 hours on a Synergy-2 plate reader. This reaction was performed in the presence of a concentration gradient of 2-AAPA ranging from 250 μM to 3.906 μM. Circles represent the mean from n=3 independent experiments with error bars showing s.d.


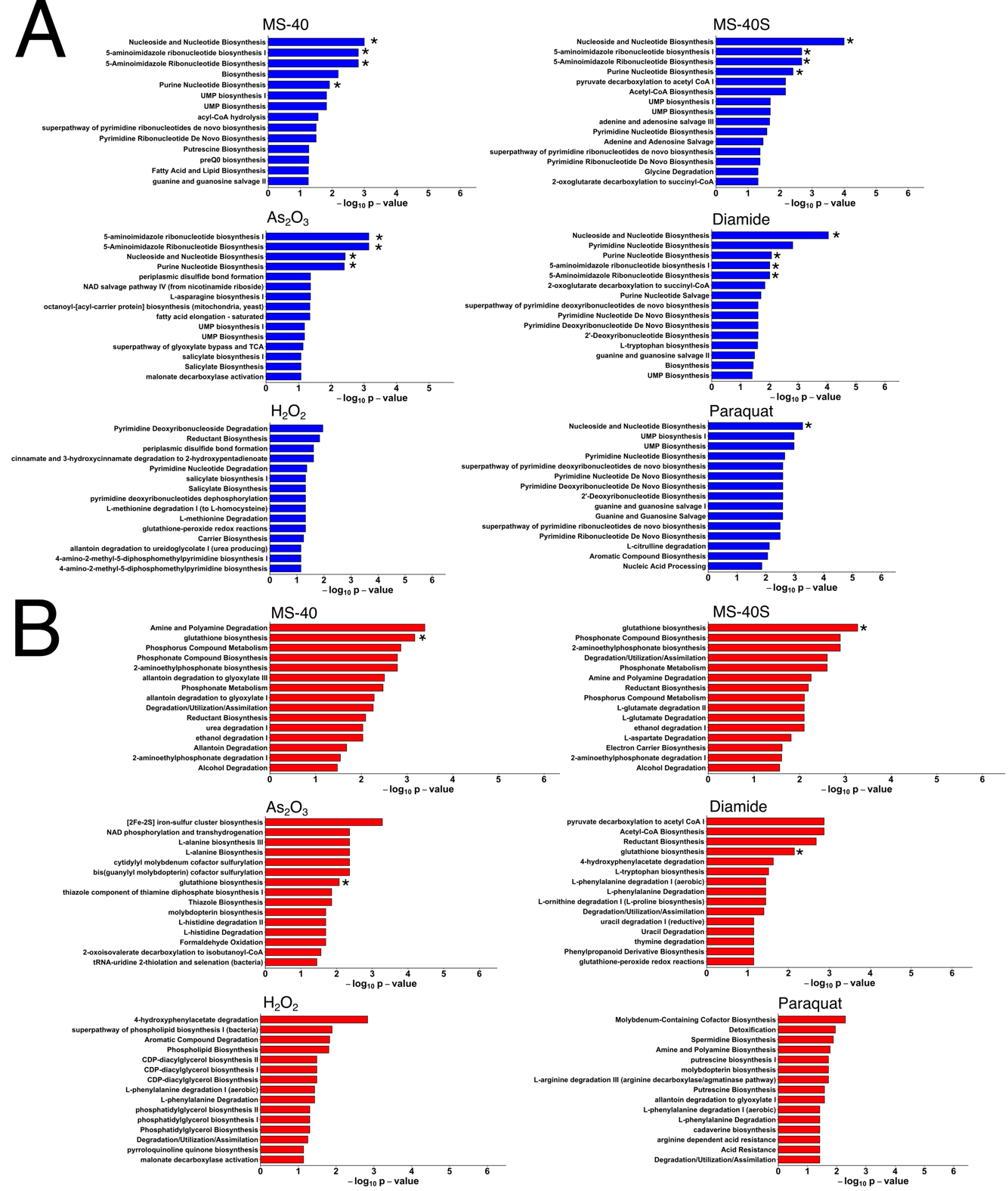


**Figure S4. BioCyc pathway enrichment with BarSeq data.** Genes with significant fitness changes, either positive (**A**) or negative (**B**), were uploaded to BioCyc Smart Tables for analysis. Pathway enrichment was done using the Fisher Exact test using the *B. cenocepacia* K56-2 database. The top 15 perturbed pathways are shown. * indicates cellular processes we explored.

**
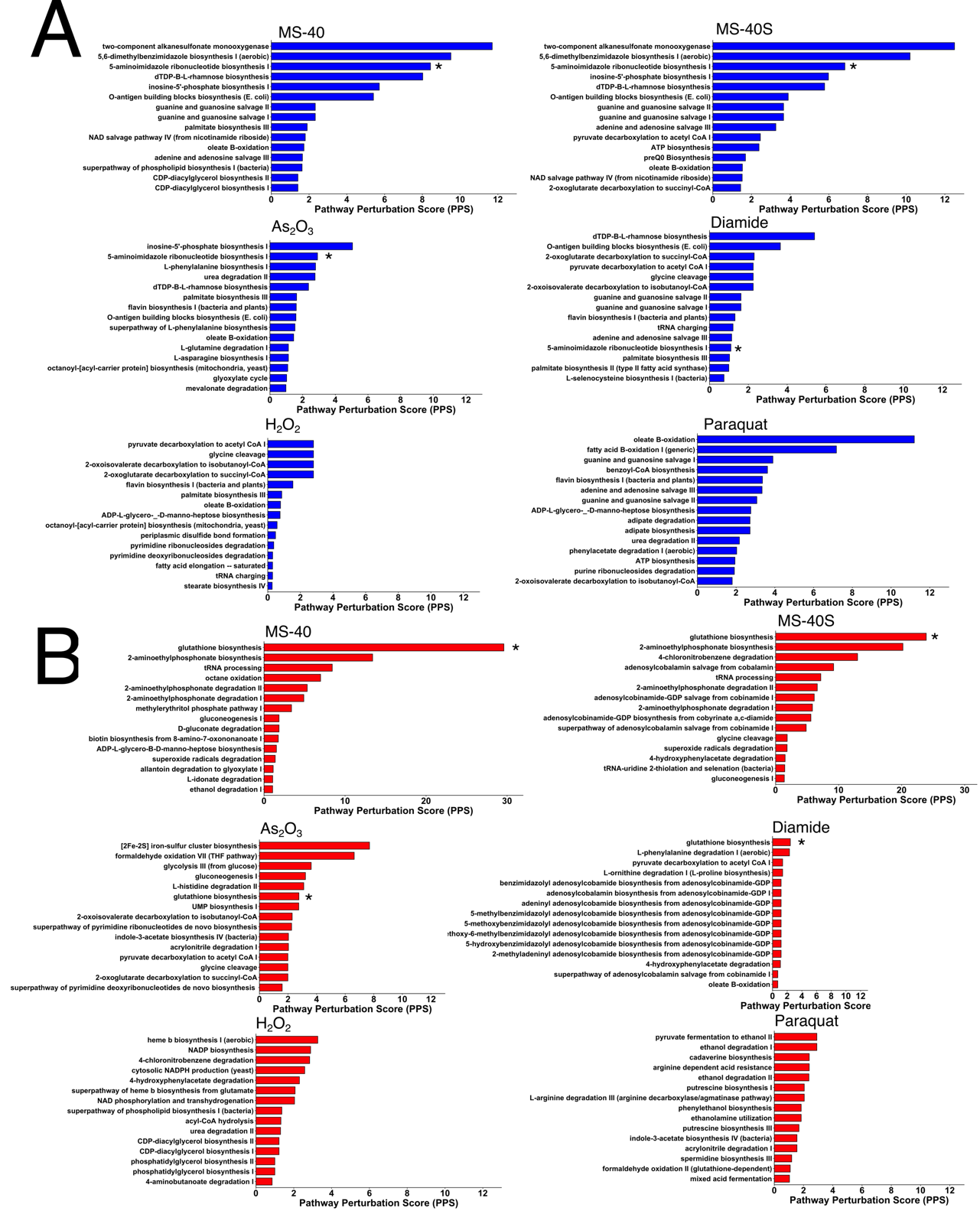
**

**Figure S5**. **BioCyc pathway perturbation score with BarSeq data.** Genes with significant fitness changes, either positive (**A**) or negative (**B**), were uploaded to BioCyc Smart Tables for analysis. Pathway perturbation score was performed from the cellular overview on BioCyc under the *Burkholderia cenocepacia* K56-2 database. The top 15 perturbed pathways are shown. * indicates cellular processes we explored.

**
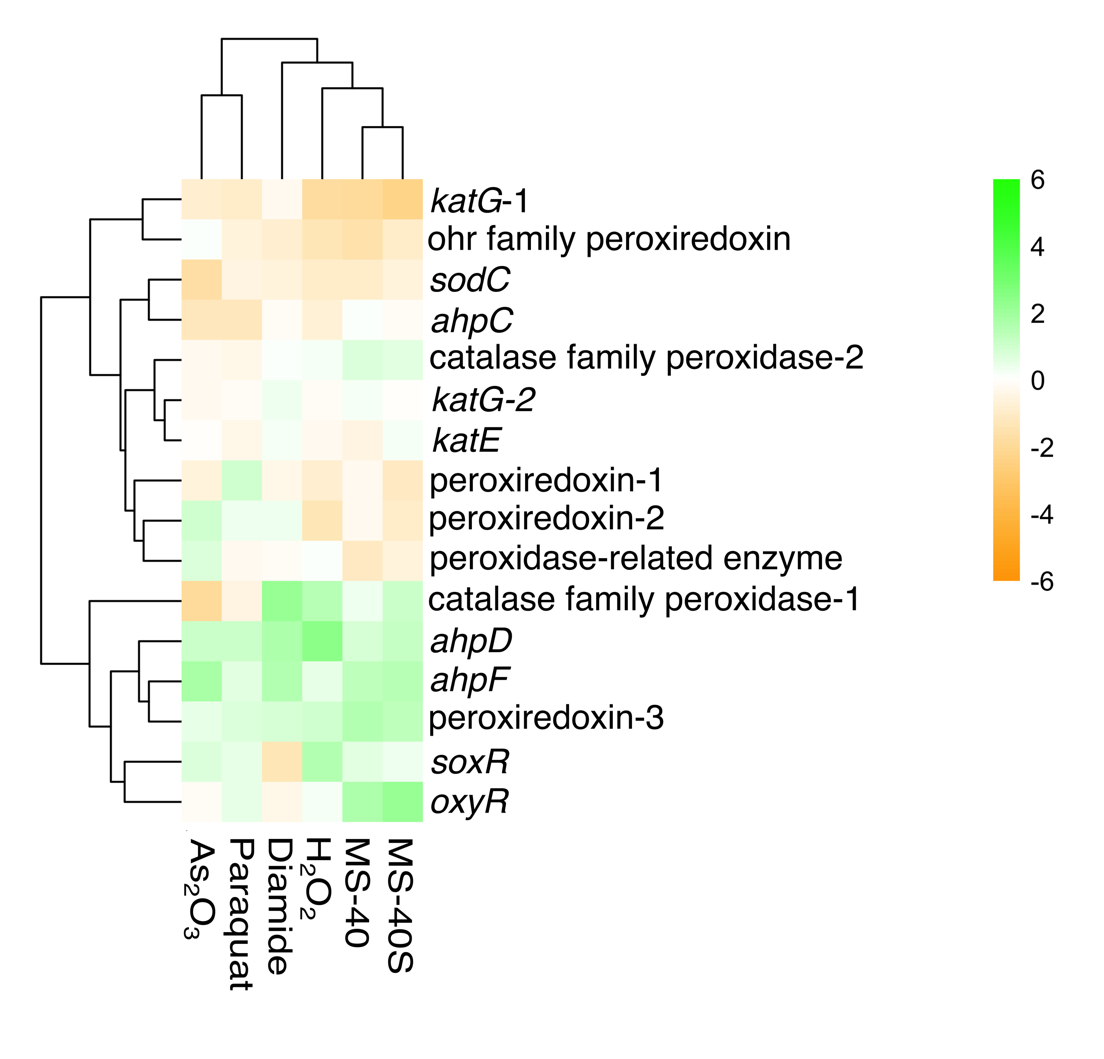
**

**Figure S6. Mutant fitness profile for ROS-related mutants in BarSeq.** Heatmap of ROS-related genes and their corresponding gene fitness values for each compound used in BarSeq. Heatmap was generated on RStudio using the pheatmap package. Green represents positive fitness; orange represents negative fitness.


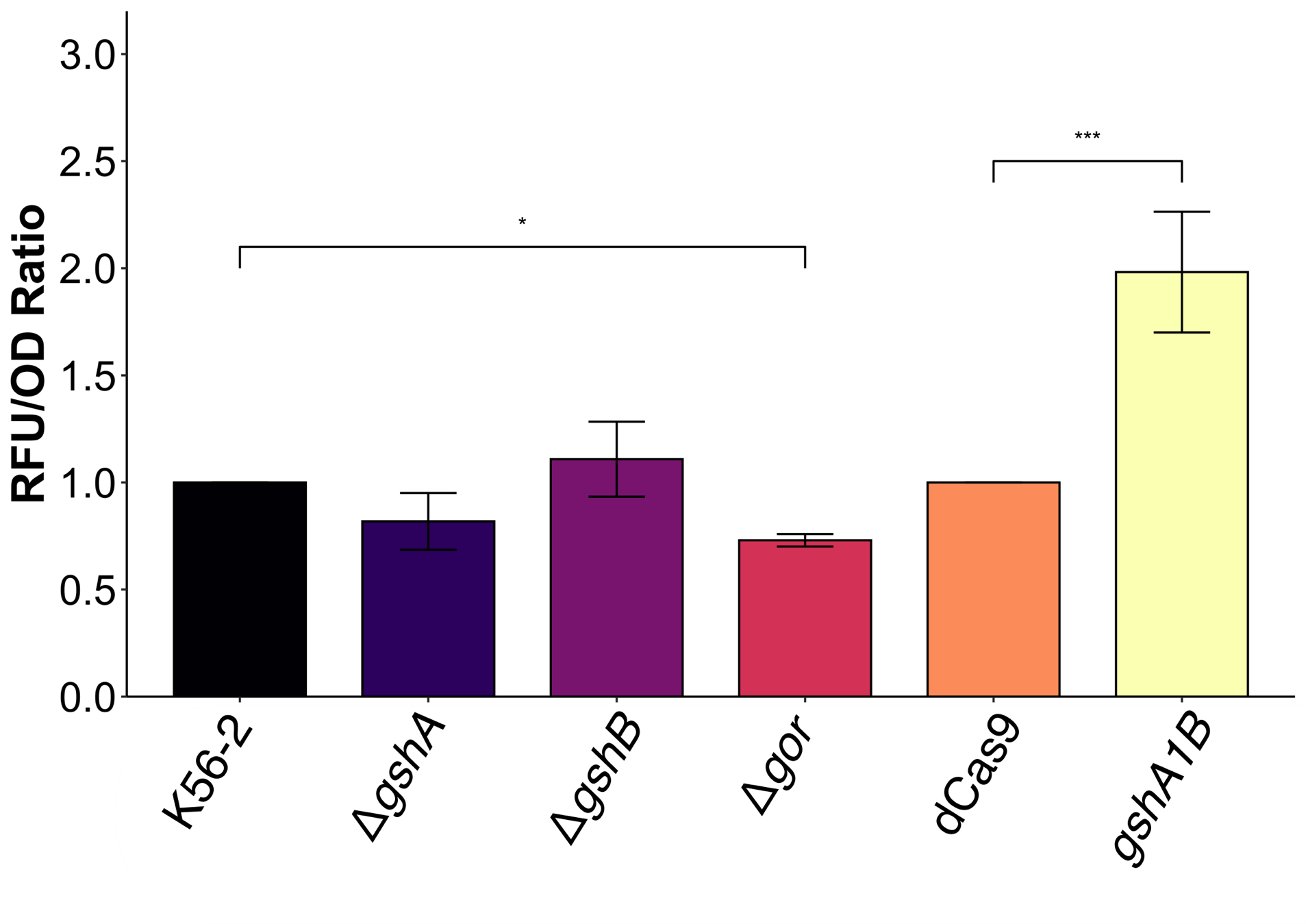


**Figure S7**. **Only the double *gshA1gshB* knockdown causes an increase in ROS.** ROS detection of glutathione deletion mutants, Δ*gshA,* Δ*gshB,* and Δ*gor* (glutathione reductase gene) and *gshA1B* CRISPRi mutant. Exponential phase cells with an approximate OD_600nm_ of 0.3 were incubated with H_2_DCFDA for 45 mins at 37˚C. Cells were then washed with PBS and added to a 96-well plate. Fluorescence was measured on a Synergy-2 plate reader. Bars represent the mean from n=3 independent experiments with error bars showing s.d. * indicates P < 0.05 and *** indicates P < 0.0001 from a one-way ANOVA and Dunnett post-hoc.


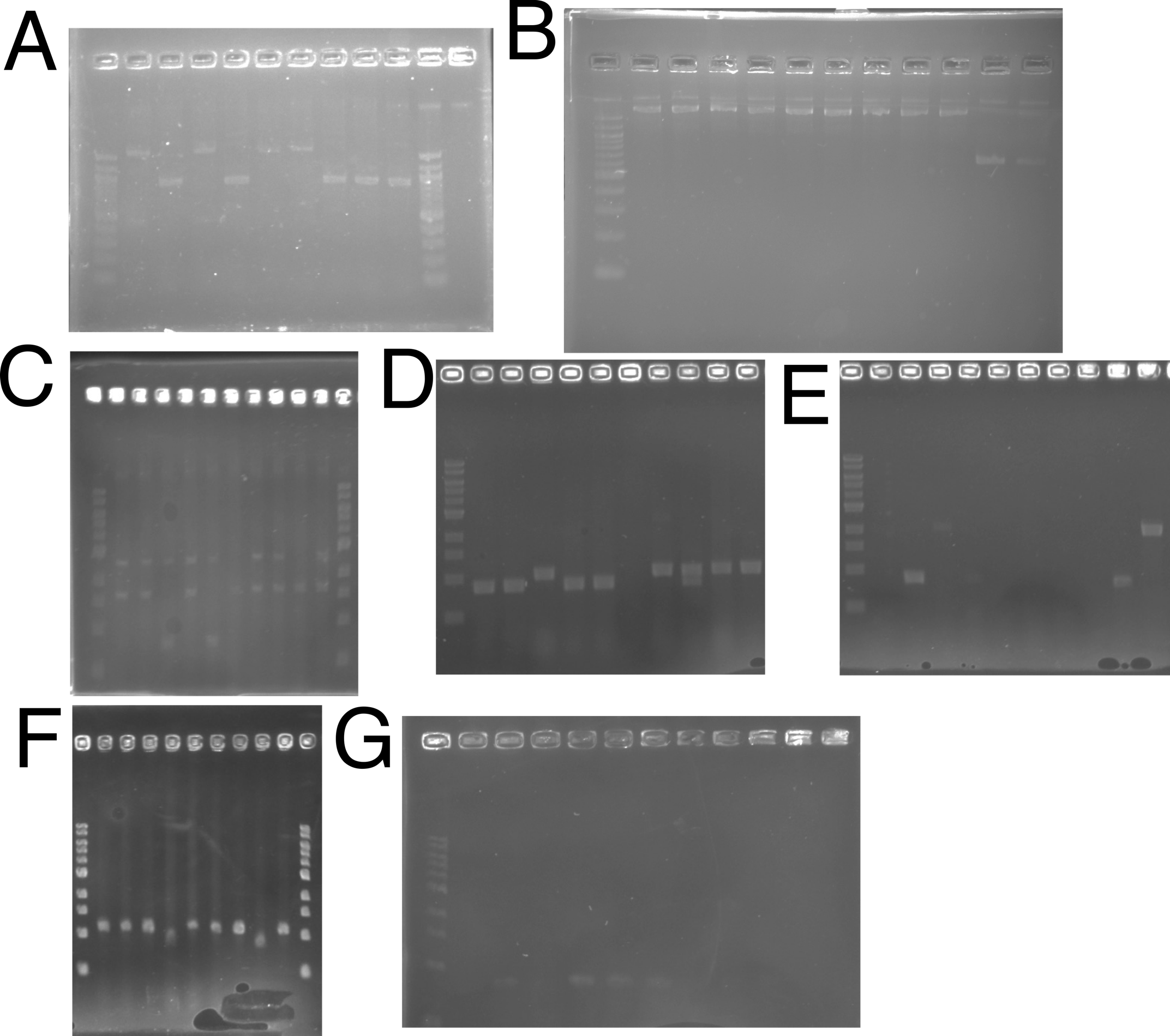


**Figure S8. Colony PCR of *oxyR* and glutathione-related gene deletions.** (A) Gene deletion of *oxyR*. WT band 2100 bp, deletion band of 800 bp. (B) Gene deletion of *gor*. WT band of 2127, deletion band of 824. (C) Gene deletion of *gshB*. WT band of 1673, deletion band of 798.

(D) Gene deletion of *grxC*. WT band of 1045, deletion band of 862. (E) Gene deletion of *gshA1*. WT band of 2655, deletion band of 1084. (F) Gene deletion of *grxD*. WT band of 1102, deletion band of 879. (G) Gene deletion of *gshA2*. WT band of 2655, deletion band of 844.

**References**

1. Aubert, D.F.; Hamad, M.A.; Valvano, M.A. A Markerless Deletion Method for Genetic Manipulation of *Burkholderia Cenocepacia* and Other Multidrug-Resistant Gram-Negative Bacteria. *Methods Mol. Biol. Clifton NJ* **2014**, *1197*, 311–327, doi:10.1007/978-1-4939-1261-2_18.

2. Hamad, M.A.; Skeldon, A.M.; Valvano, M.A. Construction of Aminoglycoside-Sensitive *Burkholderia Cenocepacia* Strains for Use in Studies of Intracellular Bacteria with the Gentamicin Protection Assay. *Appl. Environ. Microbiol.* **2010**, *76*, 3170–3176, doi:10.1128/AEM.03024-09.

3. Flannagan, R.S.; Linn, T.; Valvano, M.A. A System for the Construction of Targeted Unmarked Gene Deletions in the Genus *Burkholderia*. *Environ. Microbiol.* **2008**, *10*, 1652–1660, doi:10.1111/j.1462-2920.2008.01576.x.

4. Hogan, A.M.; Rahman, A.S.M.Z.; Lightly, T.J.; Cardona, S.T. A Broad-Host-Range CRISPRi Toolkit for Silencing Gene Expression in *Burkholderia*. *ACS Synth. Biol.* **2019**, *8*, 2372–2384, doi:10.1021/acssynbio.9b00232.

5. Hogan, A.M.; Natarajan, A.; Maydaniuk, D.T.; Léon, B.; Batun, Z.; Motnenko, A.; Palacios, A.; Bosch, A.; Cardona, S.T. Profiling Cell Envelope-Antibiotic Interactions Reveals Vulnerabilities to β-Lactams in a Multidrug-Resistant Bacterium. *Nat. Commun. Rev.* 2023, 2022.11.01.510852.

6. Darling, P.; Chan, M.; Cox, A.D.; Sokol, P.A. Siderophore Production by Cystic Fibrosis Isolates of *Burkholderia Cepacia*. *Infect. Immun.* **1998**, *66*, 874–877.
